# A Mouse Model of Oropharyngeal Papillomavirus-Induced Neoplasia Using Novel Tools for Infection and Nasal Anesthesia

**DOI:** 10.3390/v12040450

**Published:** 2020-04-16

**Authors:** Andrea Bilger, Renee E. King, Josh P. Schroeder, Jared T. Piette, Louis A. Hinshaw, Andrew D. Kurth, Ronnie W. AlRamahi, Matthew V. Barthel, Ella T. Ward-Shaw, Darya Buehler, Kristyn S. Masters, Susan L. Thibeault, Paul F. Lambert

**Affiliations:** 1 McArdle Laboratory for Cancer Research, University of Wisconsin, Madison, WI 53705, USA; bilger@oncology.wisc.edu (A.B.); mbarthel@wisc.edu (M.V.B.); etward@wisc.edu (E.T.W.-S.); 2 Department of Surgery, University of Wisconsin, Madison, WI 53705, USA; kingr@surgery.wisc.edu (R.E.K.); thibeaul@surgery.wisc.edu (S.L.T.); 3 Department of Biomedical Engineering, University of Wisconsin, Madison, WI 53705, USA; schroeder26@wisc.edu (J.P.S.); piette2@wisc.edu (J.T.P.); lhinshaw@wisc.edu (L.A.H.); akurth3@wisc.edu (A.D.K.); ralramahi@wisc.edu (R.W.A.); kmasters@wisc.edu (K.S.M.); 4 Department of Pathology and Laboratory Medicine, University of Wisconsin, Madison, WI 53705, USA; buehler2@wisc.edu

**Keywords:** mouse, papillomavirus, throat, head-and-neck, OPSCC, oropharynx, endoscopy, tumor, MmuPV1, anesthesia

## Abstract

Human head and neck cancers that develop from the squamous cells of the oropharynx (Oropharyngeal Squamous Cell Carcinomas or OPSCC) are commonly associated with the papillomavirus infection. A papillomavirus infection-based mouse model of oropharyngeal tumorigenesis would be valuable for studying the development and treatment of these tumors. We have developed an efficient system using the mouse papillomavirus (MmuPV1) to generate dysplastic oropharyngeal lesions, including tumors, in the soft palate and the base of the tongue of two immune-deficient strains of mice. To maximize efficiency and safety during infection and endoscopy, we have designed a nose cone for isoflurane-induced anesthesia that takes advantage of a mouse’s need to breathe nasally and has a large window for oral manipulations. To reach and infect the oropharynx efficiently, we have repurposed the Greer Pick allergy testing device as a virus delivery tool. We show that the Pick can be used to infect the epithelium of the soft palate and the base of the tongue of mice directly, without prior scarification. The ability to induce and track oropharyngeal papillomavirus-induced tumors in the mouse, easily and robustly, will facilitate the study of oropharyngeal tumorigenesis and potential treatments.

## 1. Introduction

Oropharyngeal squamous cell carcinoma (OPSCC) is a cancer of the throat. The oropharynx is the part of the throat, or pharynx, that opens onto the oral cavity. It includes the tonsils, the soft palate and base of the tongue, and the posterior wall of the pharynx. Over 18,000 new cases of OPSCC are diagnosed yearly in the US, mostly in men [1]. Approximately 70% of these cases are associated with the human papillomavirus (HPV). These HPV-infected oropharyngeal cancers now outnumber cervical cancer cases in the US and constitute the largest category of HPV-associated cancers [1].

Progress has been made in understanding the function of HPV in the development of OPSCC and other head and neck cancers, using transgenic mice carrying the E6 and E7 oncogenes driven by a Keratin-14 promoter [2,3]. These studies have revealed the importance of the Notch and FancD2 pathways and elucidated the roles of E6 and E7 [3,4]. However, no genetically tractable, infection-based, whole-virus model of oropharyngeal neoplasia has been available.

A papillomavirus capable of infecting laboratory mice, MmuPV1, was identified in immune-deficient mice in India in 2010 [5]. This virus has been shown to infect both cutaneous and mucosal tissues [5,6,7], where it can cause both skin and cervical cancer in immune-competent mice—particularly after immune-suppressive UVB irradiation [7,8,9]. In the mouse, deliberate primary infection with MmuPV1 elsewhere has been shown to lead to secondary infection in the oropharynx, at the base of the tongue [10]. The oropharynx in mice and humans share key anatomical features such as the soft palate, the pharynx, the base of the tongue, and stratified squamous cell epithelium. However, mice do not have tonsils. One-third of human HPV-infected oropharyngeal tumors are nontonsillar. Of these, 77% are in the base of the tongue [11].

No model involving deliberate infection of the oropharynx has been described, perhaps due to the physical challenges involved in efficiently infecting the mouse pharynx. Anesthesia is the first hurdle. Infection of the oropharynx requires that a mouse be anesthetized. Anesthetics commonly used with mice include injectable agents such as ketamine and xylazine, generally provided intraperitoneally, and inhaled agents such as isoflurane, provided via a nose cone [12]. The disadvantages of injectables include (1) the duration of anesthesia cannot be controlled easily; (2) doses that result in 100% of mice reaching a surgical plane can be fatal in a subset of animals; and (3) commonly used injectables including ketamine are controlled substances in the US and some European countries. By contrast, isoflurane “is the animal inhalation anesthetic agent of choice for both short and lengthy procedures due to its short induction and recovery time and the reliability of its effects” [12], and it is not a controlled substance in the US or Europe. However, a major limitation to the use of isoflurane is that standard isoflurane anesthesia adapters cover the head and do not allow manipulation of the oral cavity and pharynx.

The second hurdle for oropharyngeal infection is delivering the virus to the basal cells of the pharynx, which is complicated by the pharynx’s location and structure. One common infection method involves extensive injury, using a needle or cytobrush to create wounds in the epithelium down to the basal layer of cells that the virus infects, followed by topical administration of the virus [7,8,13]. Accurate manipulation of a needle or cytobrush in the confines of the mouse throat and topical administration of the virus on the sloped surfaces are a challenge, and injury to the epithelium may be painful. A second method used in the reproductive tract involves chemical injury hours before topical administration of the virus [13]. This method is time consuming and is limited both by the throat’s topology and by the types of chemicals a mouse can safely consume or aspirate.

We have designed an anesthesia cone that braces the mouse’s head while stabilizing the upper body against any firm support. The device maintains isoflurane anesthesia while holding the mouse’s oral cavity open for manipulation. We have also repurposed an allergy testing device to infect with MmuPV1 efficiently and reproducibly, with minimal pain, in the oropharynx. These tools have allowed us to easily and reliably generate dysplastic oropharyngeal lesions, including tumors, in two immune-deficient strains of mice.

## 2. Materials and Methods

### 2.1. Animals

Animal experiments (Protocol M005871) were approved 17/7/2017 by the School of Medicine and Public Health Animal Care and Use Committee (IACUC) of the University of Wisconsin-Madison and conducted in accordance with the National Institutes of Health Guide for the Care and Use of Laboratory Animals. Mice were fed an irradiated diet (Teklad 2918) or provided with an Uniprim diet (Envigo, TD06596) as needed to combat the C. bovis infection. Both infected mice and uninfected controls were given Uniprim if either group showed signs of bacterial infection. Nude mice (Fox1^nu^) were purchased from Envigo. NOD scid gamma (NSG) mice (Nod.Cg-Prkdc ScidII2rgtmWjl/SzJ; stock 005557) were purchased from the Jackson Laboratory and bred in the breeding core (BRMS, UW-Madison).

### 2.2. High-Titer Crude MmuPV1 Virus Preparation

Virus stocks were prepared from homogenized, papillomavirus-induced warts. Warts were soaked in PBS (250 mg warts/mL) overnight at 4 °C, then homogenized (PowerGen 125; Fisher Scientific, Pittsburgh, USA). After that, the homogenate was incubated with benzonase (1 µL/mL; Sigma-Aldrich; St. Louis, MO, USA) and Triton X-100 (10 µL/mL; Sigma-Aldrich). Collagenase (~5 mg/mL; type I; Worthington, Lakewood, NJ) was added to the mixture, which was then incubated for approximately 48 h at 4 °C, followed by centrifugation for 15 min at 4255× *g*. The supernatant was collected and treated with additional benzonase (1 µL/mL) and centrifuged for 10 min at 5000× *g* at 4 °C. The supernatant containing the virus was collected, and an aliquot was treated with a viral release buffer (0.1% Proteinase K, 0.5% SDS, 25 mM EDTA) and 1 ul of additional Proteinase K at 55 °C for 30 min. The released viral DNA was then analyzed by gel electrophoresis next to DNA standards for quantification, using 0.82 ng DNA = 10^8^ viral genome equivalents.

### 2.3. Nose Cone Design and Fabrication

The “Mickey’s Space Helmet” nose cone design was encoded using an Autodesk Inventor 2019 (Autodesk, San Rafael, CA, USA), using dimensions taken from 6- to 10-week-old mice. The window for oral manipulation is 15.75 × 16 mm. The adapter is compatible with an anesthesia tube with an inside diameter of 2 cm. Silicone o-rings are used to keep the mouth open via the teeth, including a 1 mm cross-section, 2.5 cm diameter silicone o-ring (“135 Pcs 1 mm Wire Diameter Silicone Rubber O-ring Seals Rings VMQ Rubber Temperature Resistant Assortment Set,” zenlis4) for the mandibular incisors, and a 1 mm cross-section, 1.5 cm diameter silicone o-ring for the maxillary incisors. Mickey’s Space Helmet is available for purchase from Jeffery Consulting, LLC (https://jefferyconsulting.com/).

### 2.4. Anesthesia and Infection

Mice were anesthetized using isoflurane provided via Mickey’s Space Helmet. A Greer Pick (Stallergenes-Greer, London, UK) was plunged into the virus stock (10^8^ to 8 × 10^9^ genome equivalents/µL) in a microfuge tube and used to mix the virus suspension. The Pick was removed by sliding along the side of the tube to remove excess virus stock. The Pick was then jabbed into a soft palate or the base of the tongue and rotated for approximately one quarter-turn.

### 2.5. Oropharyngeal Endoscopy

The endoscopy was performed using a 1.9 mm scope in an operating sheath (Karl Storz, El Segundo, USA) while mice were anesthetized in Mickey’s Space Helmet.

### 2.6. Tissue Analysis

Tongues were fixed in 4% paraformaldehyde in PBS overnight at 4 °C. Maxillae were fixed in Surgipath Decalcifier I (Leica Biosystems, Buffalo Grove, USA) overnight at 4 °C twice (with fresh decalcifier for the second incubation). Tissues were then transferred to 70% ethanol and processed. Processed tissues were embedded in paraffin. Sections (5 µm) were stained with hematoxylin (Shandon Instant Hematoxylin; #6765015, Thermo Fisher Scientific, Kalamazoo, MI, USA) and eosin (Eosin Y, #17372-87-1, mixed 10:1 with Phloxine B, #18472-87-1, Sigma-Aldrich, St. Louis, MO, USA) or analyzed immunohistochemically for the MmuPV1 L1 capsid protein or Keratin-14 as previously described [14]. Lesions arising in nude mice were evaluated by an experienced pathologist (D.B.). The significance of the differences among the degrees of dysplasia in nude and NSG mice was determined by the chi-squared statistical test, using Mstat software (Mstat version 6.5.1, McArdle Laboratory for Cancer Research; https://mcardle.wisc.edu/mstat/).

### 2.7. RNA/DNA in Situ Hybridization

The MmuPV1 nucleic acid was detected using RNAscope [15] 2.5 HD Assay-BROWN (Advanced Cell Diagnostics, Newark, CA, USA) according to the manufacturer’s instructions. Paraffin sections (5 µm) were hybridized with probes specific for MmuPV1 E6/E7. (MusPV-E6-E7; catalog no. 409771; Advanced Cell Diagnostics, Newark, CA, USA).

## 3. Results

### 3.1. A Novel Isoflurane Cone Maintains Anesthesia during Manipulation of the Throat

**Figure 1:**
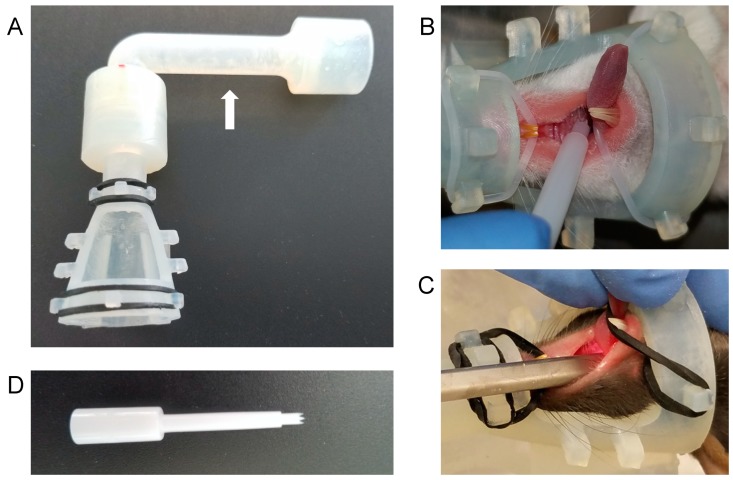
“Mickey’s Space Helmet” maintains isoflurane anesthesia during oral procedures. (**A**) The oral-access isoflurane nose cone was generated by three-dimensional (3D) printing using the bleach-resistant “Durable Resin.” The elbow adapter (white arrow) bears a small “tooth” that slides into a groove in the port at the top of the cone. This groove is at 90° to, and continuous with, a second circular groove that allows 360° rotation of the adapter relative to the cone. (**B**) The cone can be used with elastic bands (shown: silicone o-rings) to keep the mouth open via the incisors during infection with the Greer Pick. The tongue can also be restrained using the mandibular band, as shown. (**C**) The cone is also compatible with endoscopy. (**D**) The Greer Pick.

We have designed an isoflurane anesthesia cone, nicknamed “Mickey’s Space Helmet”, for procedures requiring oral access (Figure 1A–C). The design features a conical mask attached to a tubing adapter. The Space Helmet design was encoded using the Autodesk Inventor software and generated using a Formlab 3D printer. It has a large opening for oral-access manipulations, hooks for elastic bands or strings that hold the mouse’s mouth open for a hands-free operation, and an elbow adapter that can be rotated as needed during use and keeps bulky isoflurane tubing at a distance, placing the researcher in closer proximity to the anesthetized mouse. The nose cone braces the head while stabilizing the upper body against any firm support during procedures such as infection.

Elastic bands at the oral window can be used to restrain the tongue, for easy access to the oropharynx during infection or endoscopy (Figure 1B,C). Ten hooks around the window allow the cone to accommodate mouths in a wide range of sizes. In addition, the central hooks on each side of the window can be used with an elastic band as a sling under the mouse, for stability during procedures that cause significant movement. We have used the cone for the infection and/or endoscopy of mice weighing 15 to 64 g.

### 3.2. The Greer “Pick” Efficiently and Effectively Delivers Virus to the Oropharynx

The Greer “Pick” is designed for human allergy testing, to deliver antigen to the epidermis. In a comparison of skin test devices, the Greer Pick reliably elicited specific reactions (97% sensitivity) and caused the least pain [16]. The Pick holds antigen solution in a concave hollow surrounded by six sharp teeth. These teeth penetrate the outer layers of the epidermis and allow the antigen solution to enter the skin. These properties made the Pick an attractive candidate for a device that would both injure the mouse epithelium and deliver the virus to the basal cells of the epithelium that are the target of infection [17].

Our work shows that the Pick is an effective infection tool. Its 4 cm length allows it to easily reach the oropharynx when used in conjunction with Mickey’s Space Helmet (Figure 1D). No other tool is required. When the Pick is used as described in the Methods, it holds approximately 1.1 ul of virus suspension. Used to deliver MmuPV1 to nude and NSG mice, the Pick established an infection in 18 of 18 oropharyngeal sites stained for the L1 capsid protein (Figures 3 and 4).

### 3.3. Infection Yields Dysplastic Lesions, Including Exophytic Tumors, in the Soft Palate and Base of the Tongue

**Figure 2:**
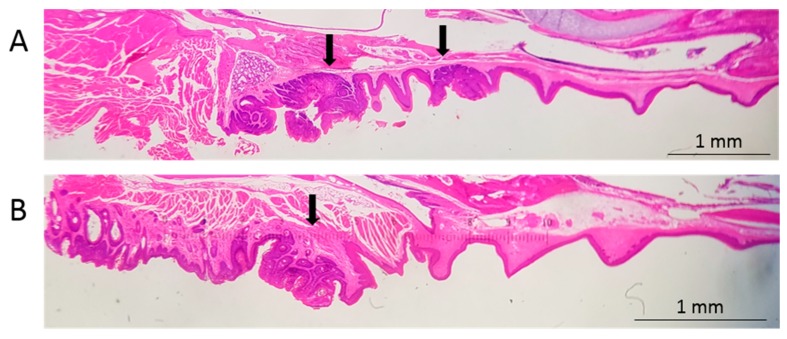
Infection of the soft palate with mouse papillomavirus (MmuPV1) elicits tumors. (**A**) A sagittal cross-section of the maxilla of a nude mouse collected 14 weeks after infection with 10^8^ genome equivalents of MmuPV1. Soft and hard palate tumors are indicated with arrows. (**B**) The maxilla of an NOD scid gamma (NSG) mouse collected 21 weeks after infection with 8 × 10^9^ genome equivalents of MmuPV1. The soft palate tumor is indicated with an arrow.

**Figure 3:**
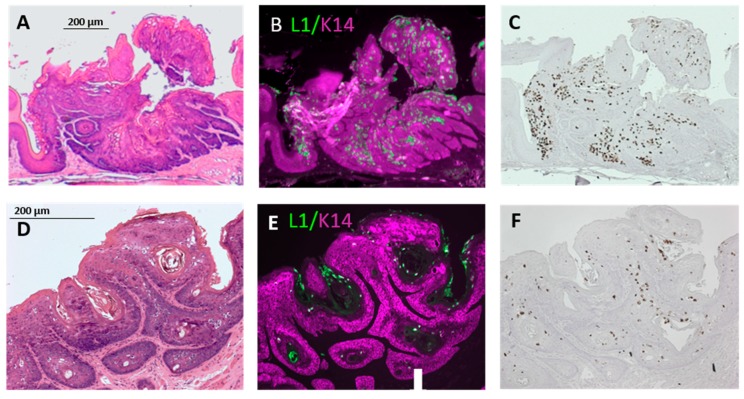
Soft palate tumors express viral capsid protein. (**A**) The soft palate tumor in Figure 2A, stained with hematoxylin and eosin (H&E). (**B**,**C**) The tumor in (**A**) after (**B**) immunohistochemical (IHC) detection of L1 capsid protein (green: “L1”) and Keratin 14 (magenta; “K14”) and (**C**) in situ hybridization with probes for MmuPV1 E6 and E7. (**D**) The tumor in Figure 2B stained with H&E. (**E**,**F**) The tumor in (**D**) after (**E**) L1 and K14 IHC and (**F**) in situ hybridization with probes for MmuPV1 E6 and E7.

Nude mice, which have compromised immunity due to a lack of T-cells, were infected in the soft palate and base of the tongue. Infection of the soft palate yielded dysplastic lesions, including noninvasive, exophytic tumors, within 14 to 21 weeks. Figure 2A shows two lesions, 14 weeks after infection, separated by apparently normal epithelium. These lesions are separated by the distance between two teeth in the Pick (1 mm) and, therefore, are likely to have been initiated by separate injuries caused by adjacent teeth in the Pick. One lesion is in the epithelium of the hard palate (characterized by bony ridges), and the other is in the epithelium covering the soft palate. Both lesions stained positive for the viral L1 capsid protein and E6/E7 nucleic acid, confirming the presence of the virus (soft palate: Figure 3A–C).

Nude mice frequently developed exophytic, noninvasive tumors in the soft palate (*n* = 5/7; Figure 2A and Figure 3A–C; Table 1). Lesions that developed over 14 to 21 weeks ranged from mildly to severely dysplastic. NSG mice, which have compromised immunity due to a lack of B-, T-, and natural killer cells, developed mild to moderately dysplastic, L1-positive, and E6/E7-positive lesions, including exophytic, noninvasive tumors, within 21 weeks of infection of the soft palate (*n* = 2/3; Figure 2B and Figure 3D–F; Table 1). The severity of the dysplasia seen in nude and NSG mice did not differ significantly (*p* > 0.06).

**Figure 4:**
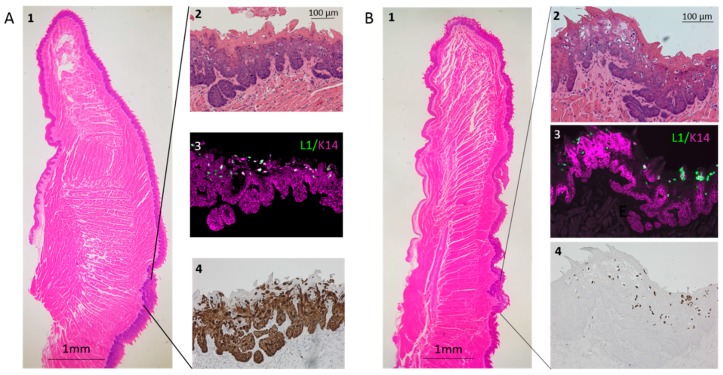
Infection of the base of the tongue with MmuPV1 elicits dysplastic lesions that express viral protein. Lesions developed at the targeted infection site approximately 2/3 of the distance from the rostral tip to the caudal end of the tongue, approximately 1–2 mm caudal of the median eminence. (**A**) Panel 1: The tongue of a nude mouse collected 21 weeks after infection with 3 × 10^9^ genome equivalents of MmuPV1. Lines demark the dysplastic epithelium shown in Panel 2, stained with H&E; Panel 3, after L1 and K14 IHC; and Panel 4, in situ hybridization with probes for MmuPV1 E6 and E7. (**B**) Panel 1: The tongue of an NSG mouse collected 21 weeks after infection with 8 × 10^9^ genome equivalents of MmuPV1. Lines demark the dysplastic epithelium shown in Panel 2, stained with H&E; Panel 3, after L1 and K14 IHC; and Panel 4, after in situ hybridization with probes for MmuPV1 E6 and E7.

Infection of the base of the tongue in nude mice induced moderately to severely dysplastic lesions that express L1 and are positive for E6/E7 nucleic acid (Figure 4A, Panels 1–4). The robust staining with E6/E7 probe suggests high expression of this transcript. In contrast to the soft palate, the tongue did not develop exophytic, papillate tumors. NSG mice also developed L1-positive E6/E7 nucleic acid-positive, dysplastic lesions at the base of the tongue (Figure 4B, Panels 1–4). These lesions showed mild dysplasia after 10 weeks and mild to moderate dysplasia after 21 weeks. The severity of the dysplasia seen in nude and NSG mice did not differ significantly (*p* > 0.47). Lesions also developed at sites that were not deliberately infected, including multiple carcinomas in situ of the outer cheek and a carcinoma of the rostral tip of the tongue.

### 3.4. Longitudinal Endoscopy Reveals Morphological Changes during Tumor Development

**Figure 5:**
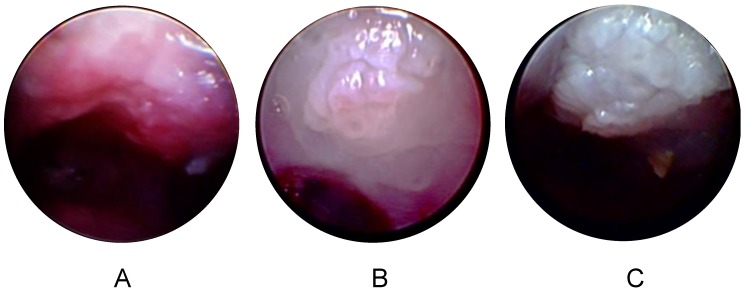
Longitudinal endoscopy reveals tumor growth. (**A**) The soft palate of an uninfected nude mouse injured with a Greer Pick and observed by endoscopy, in conjunction with Mickey’s Space Helmet, 10 weeks after injury. (**B**,**C**) The soft palate of a nude mouse observed by endoscopy 14 weeks (**B**) and 19 weeks (**C**) after infection with 3 × 10^9^ genome equivalents of MmuPV1.

We performed endoscopy to track the development of oropharyngeal tumors, taking advantage of Mickey’s Space Helmet to provide anesthesia (Figure 1C). Figure 5 contrasts an injured but uninfected soft palate (Figure 5A) with a soft palate tumor that is easily visible at 14 weeks (Figure 5B) and is visibly larger at 19 weeks (Figure 5C).

## 4. Discussion

We have developed a facile, robust, efficient protocol for generating and tracking papilloma-virus-infected oropharyngeal squamous cell tumors. Our protocol takes advantage of two innovations: A nose cone that maintains isoflurane anesthesia during oropharyngeal infection, and the Greer Pick skin allergy testing device that infects the oropharynx with MmuPV1 directly—without prior scarification. Using these tools, we consistently obtain dysplastic lesions in both the base of the tongue and in the soft palate, which often develops noninvasive, exophytic oropharyngeal squamous cell tumors.

Used clinically and sold in sterile packaging, the Pick is a convenient and versatile tool for infection. The nose cone we have designed, Mickey’s Space Helmet, provides oral access while supplying isoflurane, a fast-acting anesthetic. Devices that maintain nasal isoflurane anesthesia are available. However, these are intended for simple intubation of the lungs and do not stabilize the mouse or hold its mouth open; they are also bulky and therefore difficult to use for procedures requiring a biosafety cabinet; and they are relatively expensive (e.g., Mouse Endotracheal Intubation Kit, Kent Scientific, $675).

Mickey’s Space Helmet provides safe anesthesia during both infection and endoscopy. No other anesthesia device, to our knowledge, provides isoflurane while stabilizing the upper body and holding the mouth open for hands-free manipulation. This cone has been used successfully to maintain mice weighing 15 to 64 g under anesthesia, as long as desired. Mickey’s Space Helmet can be used in any procedure requiring access to the oral cavity or throat, and therefore should be useful in a variety of preclinical mouse trials—particularly those that require frequent anesthesia, such as longitudinal studies of treatment effects.

Longitudinal endoscopic imaging of oropharyngeal tumors is a significant advance in the MmuPV1 field. Lesion size, morphology, and other tissue characteristics can be assessed at multiple timepoints without sacrificing a large number of animals. We have demonstrated that Mickey’s Space Helmet facilitates visualization of tumor growth over time using white light endoscopy. In the future, our model could be extended to include narrow band imaging (NBI). NBI is a technology that filters all light wavelengths except for blue and green bands centered at 415 and 540 nm, respectively, to highlight blood vessels at various depths within the mucosa [18]. This greatly improves diagnostic accuracy and edge detection of even small HPV-associated lesions in the head and neck due to characteristic vascular patterns of papillomas and HPV+ SCC [19,20]. Thus, NBI may improve assessment of the presence and extent of tumors in our model, especially at early stages of tumorigenesis.

Standard treatment for OPSCC includes radiation and chemotherapy, which are associated with dose-related morbidities. Patients with papillomavirus-associated oropharyngeal tumors have a better prognosis than those with HPV− OPSCC [21]. HPV+ OPSCC is generally more sensitive to treatment and may respond to de-escalated treatments. However, treatment for HPV+ and HPV− OPSCC is almost identical [21]. Treatments tailored to the unique etiology of HPV+ disease can be tested using our infection-based model of papillomavirus tumorigenesis. This model can also be used to assess the effect of potential environmental factors, such as tobacco-associated carcinogens.

While HPV+ OPSCC is generally more responsive to the treatment than HPV− OPSCC, recent meta-analysis suggests that HPV+ OPSCCs differ in responsiveness depending on the particular tissue within the oropharynx where they developed [11]. The presence of HPV in some oropharyngeal lesions, including those of the soft palate, appears not to affect patient outcome. Our model of papillomavirus-induced soft palate tumorigenesis can be used to rigorously determine whether papillomavirus-specific treatments affect outcome in this part of the oropharynx.

One limitation to our model is that mice do not have tonsils, a lymphatic tissue. Lymphatic tissue may affect papillomavirus-induced oropharyngeal cancer development and patient outcome [11]; therefore, insights from mouse oropharyngeal models may not extend directly to tonsillar cancers. As in humans, however, the base of the tongue in mice is particularly susceptible to papillomavirus-associated carcinogenesis. Cladel et al. showed that secondary infections of MmuPV1 preferentially target the base of the tongue [10], and Mestre et al. recently found that greater than 90% of spontaneous SCCs in the oral and pharyngeal epithelia of HPV16-transgenic mice occur in the base of the tongue [22]. The authors found squamous-columnar transformation zone markers (Keratin-7 and p63) in the von Ebner’s salivary glands near the circumvallate papilla in the base of the tongue. These markers were also identified in dysplastic lesions (diffuse p63, scattered Keratin-7), suggesting that the base of the tongue contains a transformation zone similar to those observed in the cervix and anus [22,23]. Thus, the susceptibility of the base of the mouse tongue to HPV-induced carcinogenesis might be associated with structures known to be associated with HPV carcinogenesis in other susceptible tissues in mice and humans [23].

MmuPV1 was discovered as a cutaneous virus. It has since been shown to be capable of infecting a wide variety of tissues, including the cervix, the anus, and the oropharynx. Viral infection leads to carcinogenesis in the cervix, even in immune-competent animals such as mice of the FVB strain [24]. While deliberately infecting the oropharynx, we saw carcinogenesis in the rostral tongue and the outer cheek. This broad tropism contrasts with the HPV strains associated with neoplasia, which generally target mucosal or cutaneous tissues and rarely cross-infect [25]. Despite this difference in tropism, MmuPV1 appears to mimic many of the known features of neoplasia associated with more limited HPV strains: MmuPV1 cutaneous papillomas are classic warts, and MmuPV1-induced cervical cancer closely resembles human cervical cancer pathogenesis [5,9].

A desirable improvement to this model would be the ability to induce tumors in immune-competent strains. Such strains could be used to analyze the role of the immune system and, more generally, to analyze genes involved in oropharyngeal tumorigenesis—as most strains used for genetic analyses (e.g., FVB, B6) are immune-competent [4,26]. Additionally, the ability to study cancerous lesions, or to understand the factors needed to progress from in situ to the invasive disease, would increase the model’s versatility. Studies such as the analysis of tobacco carcinogens might reveal cofactors that can overcome immune defenses and/or lead to malignancy. Similarly, infecting immune-competent mice that carry mutations or genes known to promote papillomavirus oncogene-driven head-and-neck cancers (e.g., dominant-negative MAML, in the Notch pathway) [4] might yield an immune-competent, malignant model.

## 5. Conclusions

We have developed an infection-based mouse model of oropharyngeal tumorigenesis, using a device originally intended for allergy testing, to injure and deliver virus to the oropharyngeal epithelium, and a novel nose cone to manipulate and visualize the oral cavity and throat under isoflurane anesthesia. These novel tools allow us to easily generate and track virally infected lesions.

## Figures and Tables

**Table 1:** Weeks after infection of the tongue or soft palate in nude and NSG mice.

			Degree of Dysplasia							
	Incidence		Tongue			Soft Palate			Genome Equivalents	Duration (Weeks)
Strain	Tongue	Palate	Mild	Moderate	Severe	Mild	Moderate	Severe		
Nude	*NA*	1/1	*NA*			0	0	1	10^8^	14
Nude	6/6	6/6	0	3	3	2	2	2	3 × 10^9^	21
NSG	3/3	3/3	2	1	0	2	1	0	8 × 10^9^	21
NSG	3/3	*NA*	3	0	0	*NA*			3 × 10^9^	10

NA: Not applicable.
